# Cross-cultural adaptation and validation of the Global Physical Activity Questionnaire among healthy Hungarian adults

**DOI:** 10.1186/s12889-020-08477-z

**Published:** 2020-08-17

**Authors:** Pongrác Ács, József Betlehem, András Oláh, Barbara Bergier, Kata Morvay-Sey, Alexandra Makai, Viktória Prémusz

**Affiliations:** 1 Faculty of Health Sciences, University of Pécs, Pécs, Hungary; 2 Pope John Paul II. State School of Higher Education, Biała Podlaska, Poland

**Keywords:** GPAQ, physical activity, questionnaire, accelerometer, reliability, validity, cultural adaptation

## Abstract

**Background:**

Physical activity (PA) is an important factor among the determinants of health due to it’s protective factor and preventive role. Self-reported measures such as questionnaires are most commonly used in public health studies, but may over- or underestimate actual patterns of PA. Therefore, accelerometers are widely used to assess concurrent validity. The aim of the present study was to adapt and validate the self-administered GPAQ - Hungarian version (GPAQ-H) against accelerometer data and IPAQ-Hungarian long version (IPAQ-HL) in Hungarian healthy young adults.

**Methods:**

A cross-sectional comparative study was conducted to examine the last 7 days PA by GPAQ-H, comparing with IPAQ-HL and Actigraph GT3X accelerometer to measure concurrent validity and reliability. A convenient sample of 300 young adults was recruited in January – July 2018 at the University of Pécs, in South-Hungary, 120 participants (age 21.53 ± 1.75 years, 46.66% male) were included in the validity and reliability study.

**Results:**

Significant differences between the three instruments were found (*p* < 0.001) in all scores, except PAQs vigorous activities (*p* = 0.332) and GPAQ-H and accelerometer MVPA score (*p* = 0.424). A moderate KMO measure was found (0.538) with a significant Barlett’s test of Sphericity (279.51; *p* < 0.001). The total variance was explained as 81.10%. The reliability of the GPAQ-H instrument with all domain’s scores was 0.521 (CI 0.371–0.644). We found in all intensity scores and sitting time good reliability scores (*R* = 0.899–987, *p* < 0.001) between the baseline and follow-up (*N* = 33 random subsample). The Bland-Altman plots were showed that GPAQ-H overestimates vigorous activities by 212.75 min/week (331.82–757.42) and MVPA by 104.93 min/week (− 1016.98–807.11). A high difference, 6336.79 min/week (CI 3638.18–9035.40) was revealed regarding sitting, as GPAQ-H largely underestimated the time spent sedentary.

**Conclusions:**

The Hungarian GPAQ self-administered form showed fair to moderate validity with correlation coefficients similar to other European studies. Based on our study’s results it could be claimed that the GPAQ-H measurement tool is a valid and reliable questionnaire to measure the healthy Hungarian population’s physical activity patterns. However, our results also proved that GPAQ-H alone is not a valid and reliable questionnaire to measure sitting time.

## Background

Physical activity is an important factor among the determinants of health due to it’s protective factor and preventive role [1]. More than half of the Hungarian population is overweight and two thirds do not do sports regularly [2, 3]. Such behaviours among developed European citizens have been associated with chronic metabolic and musculoskeletal disorders such as type two diabetes, hypertension, obesity, and coronary heart disease, as well as psychological impairments and imbalanced mental health status [4–7].

The World Health Organization (WHO) guidelines and recommendations state that to maintain health, adults younger than 65 years old should perform at least 150 min of moderate intensity physical activity or at least 75 min of vigorous intensity physical activity throughout the week [8–10]. In this case physical activity (PA) has been defined as “*any bodily movement produced by skeletal muscles that results in energy expenditure*”. The main domains of PA are work, active transportation and leisure time activities. According to intensity, moderate (4 MET) and vigorous activities (8 MET) can be classified and walking activities should be also distinguished (multiplied by 3.3 MET) [11, 12].

The monitoring techniques are useful to examine the population’s activity and determine lifestyle trends. Self-reported measures such as questionnaires are most commonly used in public health studies because of the low costs, minimal burden, easy implementation, and valuable information. However, completing a self-administered PA questionnaire could be difficult to understand for participants, may induce bias, and thereby may over- or underestimate actual patterns of PA. Therefore, accelerometers are a widely used method to assess concurrent validity of PA questionnaires [13].

At the end of the twentieth century the International Physical Activity Questionnaire (IPAQ) was developed; the long form with 31 and the short form with 9 items [14, 15]. The long form has been considered too long and the short version not sufficient to analyse the physical activity patterns of the respondents. To complete and correct these deficiencies, the Global Physical Activity Questionnaire was compiled [16].

The Global Physical Activity Questionnaire (GPAQ) was developed by the World Health Organization (WHO) in 2002 and was endorsed as STEPwise Approach to the Chronic Disease Risk Factor Surveillance (STEPS). The questionnaire was constructed with special attention to the physical activity habits of the population of developing countries [17].

The first version of the GPAQ was validated in 9 countries, mostly in Asia, Africa, and South America. Based on the experience of the GPAQ v1, the GPAQ v2 was developed after minor revisions in 2005 with 16 items reflecting work, transportation, leisure time activities, and assessment of daily sitting time. GPAQ v2 was initially validated in Europe in Portugal and in Great Britain [18].

To ensure cultural adaptation of the tool, Mathews et al. developed a modified version of GPAQ according to local cultural tradition for adult women in India [13, 19]. The comparative validation study revealed significant but weak to moderate correlation between GPAQ and accelerometer data. The European validation studies showed weak to moderate correlation for moderate to vigorous PA (MVPA) [12, 20].

Furthermore, based on the study of Riviere et al. the IPAQ long version questionnaire proved to be an adaptive instrument to validate the GPAQ. These two quantitative techniques are similar as they contain the same domains (except the household activities which is not part of the GPAQ) and for this reason it is a relevant measurement tool to examine the concurrent validity [21].

The aim of the present study was to adapt and validate the self-administered GPAQ - Hungarian version (GPAQ-H) against accelerometer data and IPAQ-Hungarian long version (IPAQ-HL) in Hungarian healthy young adults.

## Methods

### Study design and sample

**Fig. 1:**
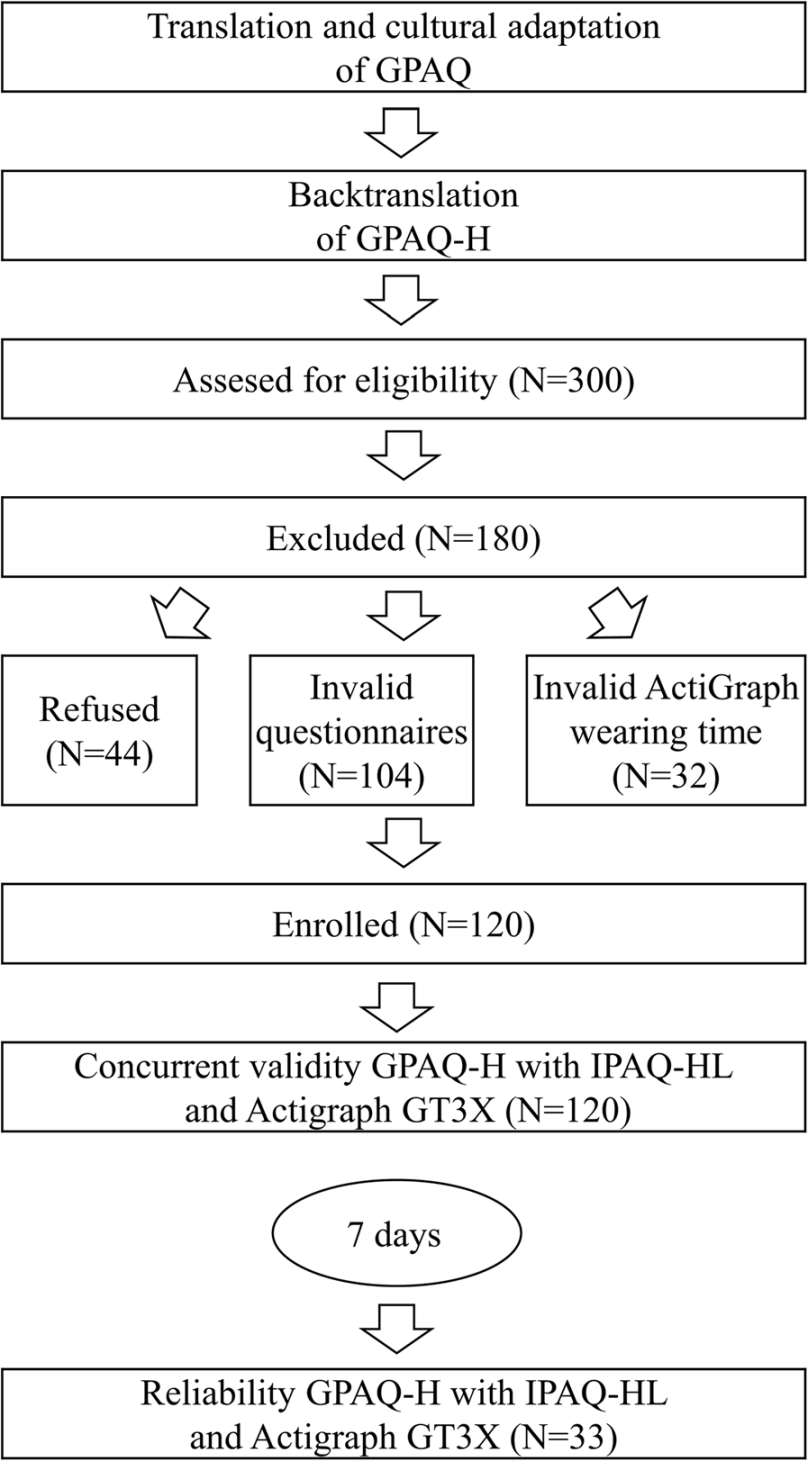
Sample flow diagram for validity and reliability study of the Hungarian Global Physical Activity Questionnaire (GPAQ-H)

A cross-sectional comparative study was conducted to examine the last 7 days PA by GPAQ-H, comparing with IPAQ-HL and ActiGraph GT3X accelerometer to measure concurrent validity and reliability. A convenient sample of 300 young adults from various faculties (Law, Medicine, Technology and Informatics and Health) of the University of Pécs in Hungary was recruited in January – July 2018. The inclusion criteria were: Hungarian-literate, absence of physical disabilities, and student status at University of Pécs. The final sample contained 120 young adults as showed on Fig. 1.

Referring to a previous study [22] which assessed the criterion validity of the GPAQ against the accelerometers, a Spearman correlation, r_s_ = 0.30 was assumed for detecting a statistically significant coefficient. To achieve a power of 80% with the level of significance at 0.05, the required sample size was 85. Considering the sample size calculation, the total sample size was designed for 100 participants per faculty. Due the high number of exclusions and refusals, the final sample size (*N* = 120) was eligible for considering the data in total, without grouping by faculty.

### Procedure and measurements

The aim of the study was explained to each participant and written informed consent was obtained before the research began by trained researchers. Participants answered a few demographic questions and anthropometric parameters were measured by OMRON B511.

Participants were asked to wear the accelerometers for a consecutive 7 days and to complete the GPAQ-H and IPAQ-HL questionnaires in self-administered form. Seven days after the first measurement was finished, participants were asked to complete the GPAQ-H and IPAQ-HL questionnaires a second time. The latter subsample contained 33 respondents.

### Physical activity outcome measures

#### Global Physical Activity Questionnaire (GPAQ)

The GPAQ version 2 was developed by the WHO, this self-administered form comprises of 16 items that measure the physical activity levels of a normal active week (7 days) of adults. The Hungarian version was developed by a scientific research group alongside native English speakers and English language experts to ensure the cultural adaptation and efficient translation of the GPAQ.

The questionnaire contains three domains of PA: work, transportation, and recreational activities. The duration and frequency of physical activity [minutes, (min/day)] were recorded in case of all three abovementioned domains.

GPAQ Analysis Guide [23] was used for scoring and data cleaning. Our study indicates data in min/week format for easier comparison with accelerometer data. Total MVPA min/week (all vigorous + all moderate activities’ mins), moderate and vigorous activities in min/week, and weekly sitting time in min/week values were calculated [23, 24].

#### International Physical Activity Questionnaire (IPAQ-HL)

The Hungarian long version of IPAQ was used to test the concurrent validity of the GPAQ-H alongside the objective measurement. The questionnaire contains 27 items formed to assess the frequency, duration, and intensity of the activities of the last 7 days. The examined domains in IPAQ-HL were work, transportation, household, leisure time activities, and time spent sitting. The data were expressed in min/week, for calculation of the different scores the scoring protocol of the questionnaire was used [14, 25]. We summarized PA in MVPA min/week, moderate and vigorous activities min/week, and sitting time in min/week also.

#### ActiGraph GT3X

Triaxial ActiGraph GT3X+ accelerometers (ActiGraph, Pensacola, FL) were used to collect data on PA with standard device initialization (sample rate of 30 Hz, 60 s epochs and normal filter option). Participants were asked to wear the devices for seven consecutive days during wakefulness on the right hip except for the following activities: water-based activities or contact sports. A run of zero counts lasting more than 60 min was defined as “non-wear time”. A minimum of 480 min of wear-time was required daily and a minimum of 5–7 days with valid wear time (where at least 1 day was a weekend day) was required for inclusion into the analysis [26]. ActiLife 6 software was used to initialize the accelerometer and to download results.

For accurate estimation of energy expenditure (EE) accelerometer outputs were converted using the algorithm Freedson Combination for Adults (Freedson 1998), with the following cut off points: sedentary (≤ 100 counts min − 1), light (101–1951 counts min − 1), moderate (1952–5724 counts min − 1) and vigorous PA (≥ 5725 counts min − 1) [27]. Participants were provided with a diary to record all non-wear time (ie when the accelerometer was removed). All recording of activities with a corresponding MET value > 1.5 were corrected for in further analysis, including activities like swimming or contact sports [28]. The average of daily moderate to vigorous physical activity (MVPA) (min / day) and sedentary behaviour (SB) (min / day) was calculated [29].

### Validity and reliability process

COSMIN checklist and Edinburgh Framework for validity and reliability were used for the validation process.

### Statistical analysis

Data were entered in Microsoft Excel and analysed using IBM SPSS 22.0 program. To present the quantitative data, mean (standard deviation, SD) and median (inter quartile range, IQR) were computed. Normality of the data was tested using Kolmogorov-Smirnov test (data was considered normally distributed if *p* < 0.05). Mann-Whitney U test and Chi-square test were calculated to measure the gender differences in PA levels. Factor analysis was conducted using principal component analysis (PCA) and varimax rotation. The Kaiser–Meyer–Olkin (KMO) index was calculated along with Bartlett’s test and anti–image correlation.

The convergent validity between the questionnaires (GPAQ-H and IPAQ-HL) and accelerometer-based measures was determined for all of the participants and examined using Spearman’s rank correlation, where > 0.40 was considered as good, 0.30–0.40 as moderate and < 0.30 as poor validity [30]. We assessed Bland-Altman plots with 95% limits of agreement to evaluate the extent of agreement between the accelerometer and the GPAQ-H and GPAQ-H and IPAQ-HL. To measure the internal consistency reliability, Cronbach Alpha was calculated. Intraclass correlation coefficient (ICC) was used for test retest reliability analysis of the GPAQ-H, where above 0.75 means were interpreted as good, 0.50–0.75 as moderate and lower means as poor reliability [11, 24]. Confidence interval of 95% was applied, and *p* value of < 0,05 was considered statistically significant.

## Results

A total of 120 young adults were included in the validity and reliability study. Average age of the participants was 21.53 ± 1.75 years. The main characteristics of the sample were showed in Table 1. The female and male participants were differed by anthropometric measures (body fat, muscle, visceral fat, waist circumference) as it was previously assumed.

**Table 1:** Characteristics of the sample in the adaptation and validation of the Hungarian version of the Global Physical Activity Questionnaire

N	Total		Male		Female		p
	120		56		64		
	Mean	SD	Mean	SD	Mean	SD	
Age (years)	21.53	1.75	21.71	1.94	21.38	1.57	0.470
BMI (kg/m^2^)	23.75	3.81	23.99	2.69	23.53	4.59	0.158
Body fat (%)	27.21	12.05	21.86	14.25	31.93	6.97	< 0.001
Muscle (%)	34.00	6.65	39.94	4.24	28.76	2.90	< 0.001
Visceral fat	4.77	2.43	5.62	2.51	4.02	2.10	< 0.001
Hip circumference (cm)	100.16	9.09	102.00	7.93	98.53	9.79	0.043
Waist circumference (cm)	78.82	13.40	85.15	8.25	73.31	14.60	< 0.001
Place of living – urban (N,%)	96	80.00	47	83.93	49	76.56	0.314
Practising sport regularly^a^ (N,%)	63	52.50	39	69.64	24	37.50	0.071
Good/very good SRH (N,%)	81	67.50	39	69.64	42	65.63	0.393

^a^3 times or more in a week

*SRH* self-reported health status

Comparing the data of the three measurements, we found significant differences between the two subjective instrument in moderate (*p* < 0.001), MVPA activities (*p* < 0.001), and sitting time (*p* < 0.001), but vigorous activities do not differ significantly (*p* = 0.332). GPAQ-H and accelerometer data showed significant differences in all of the marked scores: vigorous (*p* < 0.001) and moderate activities (*p* < 0.001) and sitting time (*p* < 0.001) except the MVPA scores (*p* = 0.424).

Analysis of the three instruments showed gender differences only in case of vigorous activities in GPAQ questionnaire (*p* = 0.046) and accelerometer data (*p* = 0.048), while sitting time accelerometer data showed significant difference among female and male participants (*p* = 0.018). (Table 2).

**Table 2:** Physical activity patterns of the sample based on accelerometer, self-administered IPAQ-HL, and GPAQ-H questionnaires

	Male (*N* = 56)					Female (*N* = 64)					p
	Mean	SD	Median	Percentiles		Mean	SD	Median	Percentiles		
				25	75				25	75	
**Accelerometer**											
Moderate min/week	332.94	158.11	332.00	232.71	438.17	343.51	115.64	341.42	255.88	413.50	0.784
Vigorous min/week	9.53	22.61	0.00	0.00	0.88	3.91	13.58	0.00	0.00	0.00	0.048*
MVPA min/week	342.71	164.06	343.00	232.71	438.17	347.98	114.06	347.75	265.17	417.83	0.873
Sitting time min/week	9037.68	437.12	8953.58	8744.00	9302.08	9167.18	291.27	9187.00	8968.83	9393.63	0.018*
**IPAQ-HL**											
Moderate min/week	328.05	316.81	215.00	100.00	487.50	367.19	343.65	250.00	123.75	588.75	0.497
Vigorous min/week	325.67	298.14	259.75	96.39	451.05	201.88	242.35	129.70	9.26	291.45	0.017*
MVPA min/week	647.39	509.74	471.18	270.75	884.50	567.36	482.86	423.68	225.25	828.25	0.364
Sitting time min/week	2618.75	1099.95	2610.00	2055.00	3435.00	2571.69	1016.49	2640.00	1815.00	3420.00	0.666
**GPAQ-H**											
Moderate min/week	241.07	299.09	135.00	22.50	345.00	222.50	286.11	120.00	37.50	333.75	0.779
Vigorous min/week	290.07	349.78	180.00	60.00	360.00	157.34	171.83	120.00	2.50	262.50	0.046*
MVPA min/week	531.14	539.20	345.00	143.75	697.50	379.84	381.55	285.00	90.00	551.25	0.150
Sitting time min/week	2703.13	1306.28	2520.00	1680.00	4200.00	2828.44	1326.03	2940.00	2100.00	4095.00	0.639

### Validity and reliability of GPAQ-H

#### Concurrent validity of the GPAQ-H instrument

We tested the validity of the GPAQ-H by using Spearman’s rank correlation between accelerometer and GPAQ-H and IPAQ-HL and GPAQ-H. (Table 3).

**Table 3:** Concurrent validity of the GPAQ-H comparing by accelerometer and IPAQ-HL

ActiGraph GT3X												
	Total				Male (*N* = 56)				Female (*N* = 64)			
	M	V	MVPA	SB	M	V	MVPA	SB	M	V	MVPA	SB
GPAQ-H M												
R	.185^*^	−.103	.168	−.170	.146	−.241	.099	−.053	.224	.049	.239	−.273^*^
p	**.043**	.262	.067	.063	.284	.074	.466	.698	.075	.703	.057	**.029**
GPAQ-H V												
R	.381^**^	−.109	.359^**^	−.325^**^	.430^**^	−.099	.399^**^	−.311^*^	.335^**^	−.207	.309^*^	−.270^*^
p	**<.001**	.235	**<.001**	**<.001**	**.001**	.466	**.002**	**.020**	**.007**	.101	**.013**	**.031**
GPAQ-H MVPA												
R	.290^**^	−.116	.269^**^	−.296^**^	.315^*^	−.227	.264^*^	−.203	.289^*^	−.047	.291^*^	−.340^**^
p	**.001**	.206	**.003**	**.001**	**.018**	.093	**.0497**	.133	**.020**	.710	**.020**	**.006**
GPAQ-H SB												
R	.106	−.014	.098	−.007	.135	.090	.154	−.172	.081	−.092	.056	.064
p	.249	.879	.287	.936	.320	.509	.257	.205	.525	.471	.663	.617
**IPAQ-HL**												
	Total				Male				Female			
	M	V	MVPA	SB	M	V	MVPA	SB	M	V	MVPA	SB
GPAQ-H M												
R	.504^**^	.424^**^	.541^**^	.007	.526^**^	.425^**^	.560^**^	−.265^*^	.484^**^	.454^**^	.526^**^	.262^*^
p	**<.001**	**<.001**	**<.001**	.943	**<.001**	**.001**	**. <.001**	**.048**	**<.001**	**<.001**	**<.001**	**.036**
GPAQ-H V												
R	.431^**^	.715^**^	.623^**^	−.011	.422^**^	.761^**^	.692^**^	−.189	.470^**^	.640^**^	.564^**^	.152
p	**<.001**	**<.001**	**<.001**	.908	**.001**	**<.001**	**<.001**	.163	**<.001**	**<.001**	**<.001**	.230
GPAQ-H MVPA												
R	.527^**^	.603^**^	.644^**^	−.013	.555^**^	.653^**^	.723^**^	−.293^*^	.519^**^	.546^**^	.571^**^	.251^*^
p	**<.001**	**<.001**	**<.001**	.892	**<.001**	**<.001**	**<.001**	**.028**	**<.001**	**<.001**	**<.001**	**.046**
GPAQ-H SB												
R	−.046	.056	−.034	.378^**^	−.052	−.098	−.128	.403^**^	−.051	.207	.044	.345^**^
p	.615	.546	.710	**.000**	.704	.473	.347	**.002**	.689	.101	.730	**.005**

*GPAQ-H*Global Physical Activity Questionnaire – Hungarian Version, *IPAQ-HL* International Physical Activity Questionnaire – Hungarian Long Form, m/v: minute/week, *M* moderate, *MVPA* total moderate to vigorous physical activity, *SB* sedentary behaviour, *V* vigorous

#### Content validity of the GPAQ-H instrument

The content validity of the questionnaire was examined by factor analysis using principal component analysis with varimax rotation. A moderate KMO measure was found (0.538) with a significant Barlett’s test of Sphericity (279.51; *p* < 0.001). The total variance was explained as 81.10%. We found five factors as follows: Factor 1 work vigorous activities, work and leisure time together (24.45% of variance), Factor 2 moderate leisure time activities (15.99% of variance), Factor 3 moderate work time activities (15.65%), Factor 4 active transportation (15.10%), and Factor 5 sitting time (9.91% of the variance).

#### Internal consistency and test retest reliability of the GPAQ-H

The reliability (Cronbach Alpha) of the GPAQ-H instrument with all domain’s scores was 0.521 (confidence interval (CI) 0.371–0.644). In our study after 7 days of the first data collection a subsample of our baseline sample completed the GPAQ-H measurement tool. We found in all intensity scores and sitting time (moderate, vigorous, MVPA and sitting time) good reliability scores (*R* = 0.899–987, *p* < 0.001) between the baseline and follow-up scores.

**Fig. 2:**
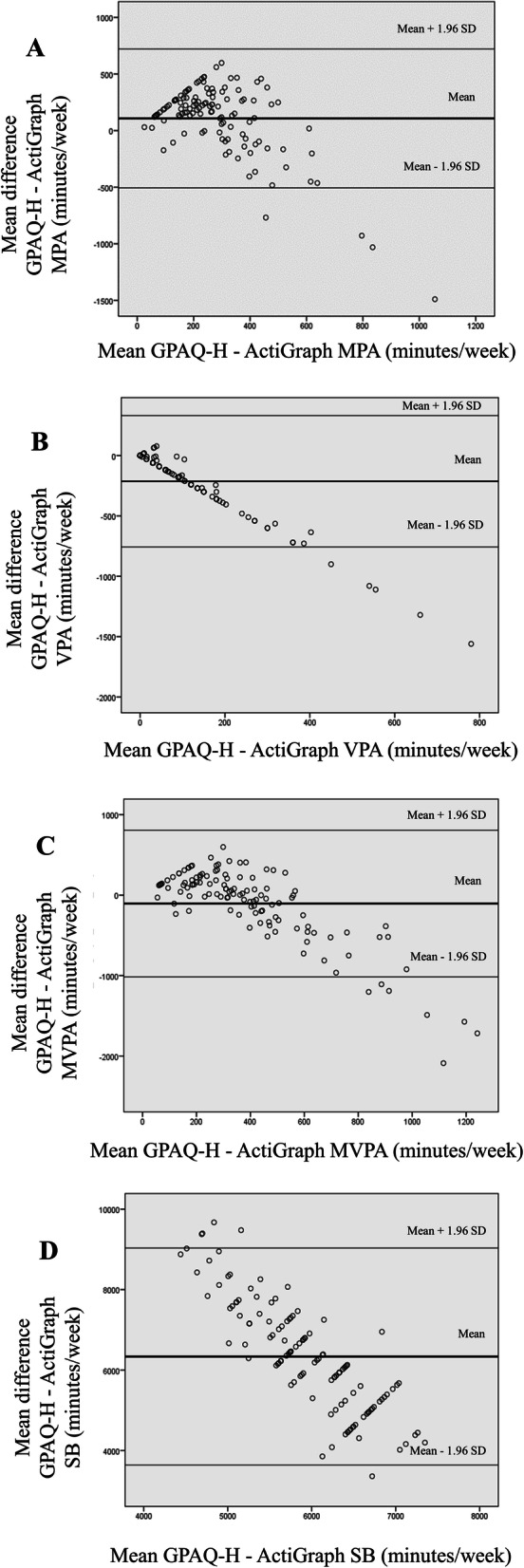
Bland Altman plots between GPAQ-H and accelerometer (95% Limits of agreement)

Bland Altman plots demonstrated differences between the GPAQ-H and accelerometer mean values (Fig. 2). The plots showed that GPAQ-H overestimates vigorous activities by 212.75 min/week (331.82–757.42) and MVPA values by 104.93 min/week (− 1016.98–807.11). A high difference, 6336.79 min/week (CI 3638.18–9035.40) was revealed regarding sitting, as GPAQ-H largely underestimated the time spent sedentary. Furthermore, the plots indicated wide limit of agreements for all examined parameters.

## Discussion

This study showed the validity and reliability of the GPAQ-H measurement tool in comparison with accelerometer and IPAQ-HL data. Our results demonstrated fair to moderate validity of the Hungarian GPAQ compared to the accelerometer data and moderate and good correlation with IPAQ-HL questionnaire. We examined the correlation between accelerometer and questionnaires according to moderate, vigorous, MVPA activities, and sitting time values. Our results are consistent with other studies according to the intensity of the correlation coefficients.

The GPAQ-H vigorous data were showed significant moderate correlation with accelerometer-moderate and accelerometer-MVPA results, but there were no significant results with accelerometer-vigorous data. The GPAQ-H moderate values did not correlate with MVPA, only with accelerometer-moderate results. The GPAQ-H MVPA showed significant correlation with moderate and MVPA accelerometer values. The GPAQ-H sitting time did not correlate with the examined accelerometer parameters. In case of the subgroup analysis our results were similar according to genders. We noticed significant difference only by vigorous activities irrespective of the measurement method (GT3X *p* = 0.048, GPAQ-H *p* = 0.046, IPAQ-HL *p* = 0.017), and by objectively measured sitting time (*p* = 0.018). Otherwise, in case of the total sample, sitting time did not show a significant correlation between questionnaire and accelerometer data, but there was a significant negative correlation between accelerometer sitting time value, the GPAQ-H MVPA (*R* = -0.296, *p* < 0.001), and vigorous values (*R* = -0.325, *p* < 0.001). The GPAQ-H and IPAQ-HL questionnaires showed moderate and good correlation and similar mean values, but the overestimation of the MVPA, moderate and vigorous activities was higher in IPAQ-HL.

In the French validation study of GPAQ, Riviere et al. applied similar study design as our research group: they measured PA patterns of staff members and students (*N* = 92, age 30.1 ± 10.7, 76.9% BMI 18.5–24.9) of the University of Lorraine, using IPAQ-LF for concurrent and ActiGraphs for criterion measures. Multiple overestimation of PA – in particular for vigorous intensity (more than tenfold) – was characteristic in case of self-reports. Regarding intensity, Riviere et al. found correlation only between vigorous activities (*R* = 0.38) and not any significant relationship between moderate activities (*R* = 0.10). Comparing total activities across all domains of GPAQ with accelerometer-moderate activity (*R* = 0.40) and with accelerometer vigorous values (*R* = 0.24), modest significance was found. They observed poor significant relationship when examining the correlation between self-reported sitting time, accelerometer-sitting time (*R* = 0.42), and accelerometer-moderate activities (*R* = -0.22). By retest, the research found poor values by moderate leisure and total PA (ICC = 0.37 and 0.58 respectively) but good or almost perfect values by total sedentary and vigorous PA at work (ICC = 0.80 and PABAK = 0.91). Comparing GPAQ and IPAQ-LF, important discrepancies were found, and the classification with level of PA was only poorly to moderately correlated by the concurrent validity (Phi coefficient 0,22–057) [21].

Mumu et al. found fair to moderate correlation between objective and subjective monitoring, still claimed GPAQ as an acceptable measure, particularly among women with higher level of education despite the under-estimation of sedentary behaviour (*R* = 0,23, *p* < 0.001) [31]. The authors explain divergence of the results by genders with PA habits, contrary of other studies [12] in favour of females. In Bangladesh – a least developed country according to United Nations classification – walking is more specific by work activities for females which is more reliably monitored with accelerometers than upper-body motions of males during intensive farming or carriage of heavy loads e.g. swimming or cycling. 60% of the sample in the study by Mumu et al. belongs to the rural population – in our study, 96% of the sample belongs to urban population. This difference may be behind more equal PA habits between genders.

Meeting PA guidelines but being highly sedentary for the rest of the days is also an emphasized risk factor [32]. Chu et al. negotiated sedentary behaviour measures, using a domain-specific Adult Sedentary Behaviour Questionnaire (ASBQ) and the Global Physical Activity Questionnaire’s (GPAQ) single-item sitting question against triaxial ActiGraph wGT3X-BT accelerometers. They found significant correlation between accelerometer and GPAQ in sedentary time, while the GPAQ under-estimated the time spent sedentary. However, moderate to good test-retest reliability (*R* = 0.74, ICC 0.62–0.82) was presented [22].

Measurement of inactivity proved to be doubtful in other studies as well. Cleland et al. found poor correlation with daily sitting time in minutes (*R* = 0.187), and reported that those people who are more sedentary were less likely to under-report their level of SB. The authors stated that this questionnaire could not be considered as a valid tool to measure sitting time [12]. They postulated both long (*R* = 0.33) and short (*R* = 0.34) form of IPAQ with higher sitting items more appropriate referring to a previous research [33]. We observed the same tendency in our study and we hypothesise that this may be due to the context of health literacy and health behaviour of participants. Describing perceived levels of activities is difficult, not only in case of SB but also regarding MVPA. Cleland et al. found only moderate correlation between the objective and subjective measurement (*R* = 0.484) of MVPA (10).

However, GPAQ was originally designed to be interviewer-administered by the WHO. Yet, similarly to many authors, we also decided to record the questionnaires in self-administrated form. The way of query did not justify bias or discrepancy in prior examinations. In the study of Chu et al. data with self-administration were not weaker than with interviewer-administration, yet they found only fair-to-moderate correlations for moderate-to-vigorous physical activity (*R* = 0.30, *R* = 0.46 respectively). Strongest correlations were observed for vigorous-intensity activity (self-report *R* = 0.38, with interviewer *R* = 0.52). Bias were illustrated with Bland-Altman plots toward overestimation of higher levels of vigorous- and moderate-intensity activities, and underestimation for lower levels PA, parallel to similar studies in general. Reliability for MVPA revealed moderate correlations (self-report *R* = 0.61, with interviewer *R* = 0.63). To reduce bias in the GPAQ measurements they advised to incorporate accelerometers, particularly by the measurement of different intensity PA (A. H. Chu, Ng, Koh, & Muller-Riemenschneider, 2015).

Wanner et al. measured the validity of GPAQ in European context. They found significant results as other Western countries, like fair-to-moderate validity of the GPAQ questionnaire. The range of the overestimation of the GPAQ was between 2.8–4.2 times, which mean that GPAQ results are notably higher than the accelerometer data. Total activities showed fair correlation between GPAQ and the accelerometer (*R* = 0.22), but the MVPA showed weak correlation (*R* = 0.11), while vigorous activities were moderately correlated with accelerometer (*R* = 0.46) like sitting time (*R* = 0.47). The results of the Wanner et al. study showed significant difference between gender, where male participants were more likely to overestimate their vigorous activities [20]..

The reason for overestimation of time spent with MVPA may also be in relation with the lack of appropriate knowledge on adequate value of health enhancing physical activity and the perception of importance of physical activity [34]. Besides, a better understanding of the questionnaires could help to receive more accurate results. Cleland et al. also found higher validity in higher-income countries due to higher education levels [12].

Contrary to the above results, Laeremans et al. compared GPAQ results with another wearable sensor (SenseWear) in a multi-centre (Antwerp-Barcelona-London) study and demonstrated significantly lower (*p* < 0.05) time and energy expenditure (MET) in GPAQ MVPA than with SensWear. Nevertheless, the study found significant correlation (0.45–0.64) between these variables. They reported also unusual findings in relation to SB, which did not differ by various instruments, yet it was poorly correlated (R < 0.25). However, vigorous PA values showed high similarity (R > 0.59) [17].

To improve the validity of GPAQ data, Metcalf et al. highlighted the utility of a Mean Squared Prediction Errors model for calibration. In this study data were collected in Ottumwa (IA, USA) and accelerometer data were predicted with a multiple regression model regarding gender, age, GPAQ PA domains by intensity and SB as covariates. The authors found weak correlation between self-reported and objectively measured data in Outcome Matching Model (R^2^ = 0.025–0.177), but using Break Factor Cut-offs the Final Calibration Model showed considerable improvement (R^2^ = 0.097–0.364). In both models the proportion of variance explained of vigorous PA was the highest and SB the lowest. Mean Squared Prediction Errors reduced from 66.4–98.3% to 61.3–98.6% [19]. Majority of these studies show that, compared to other PA questionnaires, the GPAQ is more appropriate for monitoring physically active people and activities with higher energy expenditure.

While this current study focused on young adults, aging people belonging to a high risk population, should be negotiated with particular attention. Results from the GPAQ study of Hamrik et al. (carried out in the Czech Republic, a region which is socio-economically similar to Hungary) highlighted that more than 60% of the studied population across all ages could be described as sedentary, but the levels of PA decrease more with age (OR/95% CI1.011/1.005–1.017; F^age^ = 8.002, *p* < 0.001). They reported the highest level of sedentary behaviour over 65 years [35]. These facts indicate the need for repeated monitoring of PA through the lifespan. While GPAQ is not suitable for reporting changes in individual PA habits, it appears to be a valid tool for monitoring national strategies for PA promotion, especially for MVPA [12].

### Limitations

However, the Hungarian results confirmed that GPAQ is a valid and reliable tool to examine the Hungarian population’s physical activity level, it should be borne in mind that self-administration of data can be a challenge [20], GPAQ as other subjective measurements based on self-reported data, can over or underestimates values of the physical activity level [36]. On the other hand, opposite to the self-report measures accelerometer do not register the cycling, contact sport and swimming time and it was not wearing all day.

GPAQ is a widely used tool to measure the effect of interventions at population- or community level, but it is not an efficient tool to measure changes in an individual’s physical activity [12].

## Conclusion

The Hungarian GPAQ self-administered form showed fair to moderate validity with correlation coefficients similar to other European studies. Based on our study’s results it could be claimed that the GPAQ-H measurement tool is a valid and reliable questionnaire to measure the healthy Hungarian population’s physical activity patterns. The validity is fair to moderate but acceptable, like other similar Europeans studies. Our results also proved that GPAQ-H alone is not a valid and reliable questionnaire to measure sitting time.

## Data Availability

The dataset supporting the conclusions of this article is available from the corresponding author on reasonable request.

## Abbreviations

ASBQ: Adult Sedentary Behaviour Questionnaire
BMI: Body mass index
CA: Cronbach’s Alpha
ICC: Intraclass correlation coefficient
GPAQ: Global Physical Activity Questionnaire
IPAQ: International Physical Activity Questionnaire
IQR: Inter quartile range
KMO: Kaiser–Meyer–Olkin
MET: Metabolic equivalent of task
MVPA: Moderate to vigorous physical activity
PAQs: Physical activity questionnaires
PCA: Principal component analysis
SB: Sedentary behaviour
SD: Standard deviation
SRH: Self-reported health
WHO: World Health Organization
STEPS: STEPwise Approach to the Chronic Disease Risk Factor Surveillance
